# Cannabis consumption and problematic internet use: analysis of a free-text response collected through a self-reported online questionnaire

**DOI:** 10.1186/s40359-026-04419-3

**Published:** 2026-03-24

**Authors:** Juliette St-Onge, Magaly Brodeur, Andrée-Anne Légaré, Amélie Deschamps, Anne-Marie Auger, Natalia Muñoz Gómez, Catherine Hudon, Didier Jutras-Aswad, Anders Håkansson, Virginie Parent, Adèle Morvannou

**Affiliations:** 1 Department of Family Medicine and Emergency Medicine, Faculty of Medecine and Health Sciences, Université de Sherbrooke, 3001, 12e Avenue Nord, Sherbrooke, QC J1H 5N4 Canada; 2 Centre de recherche du Centre hospitalier universitaire de Sherbrooke, 12e Avenue N Porte 6, Sherbrooke, QC J1H 5N4 Canada; 3 Department of Community Health Sciences, Université de Sherbrooke, 3001, 12e Avenue Nord, Sherbrooke, QC J1H 5N4 Canada; 4 Addiction Services, Université de Sherbrooke, 3001, 12e Avenue Nord, Sherbrooke, QC J1H 5N4 Canada; 5 Department of Psychiatry and Addiction, Université de Montréal, 2900, boul. Édouard-Montpetit, Montreal, QC H3T 1J4 Canada; 6 Centre hospitalier de l’Université de Montréal (CHUM) Research Center, 1000, rue Saint-Denis, Montreal, QC H2X 0C1 Canada; 7 Clinical Addiction Research Unit, Faculty of Medicine, Lund University, Entrégatan 7, 222 42 Lund, Sweden; 8 Centre intégré universitaire de santé et de services sociaux de l’Estrie, Centre hospitalier universitaire de Sherbrooke, 375 rue Argyll , QC J1J 3H5 Sherbrooke, Canada

**Keywords:** Cannabis, Internet, Qualitative, Behavioral addiction

## Abstract

**Background:**

Cannabis, the most consumed illicit drug globally, has been legal for non-medical use in Canada since 2018. Problematic internet use is considered a behavioral addiction involving excessive online activities (e.g., social media, video watching), which can cause social isolation, family issues, psychological distress, and even suicide ideation. Studies mainly conducted with adolescents indicate a link between cannabis use disorder and problematic internet use.

**Methods:**

This exploratory study with a qualitative component aims to investigate the experiences of adult cannabis users in Quebec regarding their internet use and cannabis consumption. Data were collected via an open-ended question in a self-reported online questionnaire assessing the participants’ perceived impact of cannabis consumption and internet use on their lives. The 1,388 responses to the open-ended question were subjected to a thematic analysis.

**Results:**

The analysis yielded four main themes: (1) no impact and no link, (2) link between cannabis consumption and internet use, (3) psychological and physical health and (4) lifestyle habits. Some participants noted a direct association between cannabis use and increased online activity, including online pornography use. The participants experienced both positive and negative effects from cannabis consumption and internet use. Many reported benefits, such as relaxation, positive emotions, improved mental skills, and pain relief, while others experienced negative impacts, such as increased anxiety, procrastination, social isolation, and impaired cognitive functions. Additionally, these behaviors affected aspects of their daily lives, including finances, sleep, eating habits, and relationships.

**Conclusions:**

This study provides insights into the participants’ experiences and reflects the aspects they considered essential to share regarding cannabis consumption and internet use.

**Clinical trial number:**

Not applicable.

**Supplementary Information:**

The online version contains supplementary material available at 10.1186/s40359-026-04419-3.

## Introduction

According to the World Health Organization [1], cannabis is the most cultivated, trafficked, and used illicit drug worldwide. In 2018, non-medical cannabis consumption and the regulated sale of cannabis were legalized in Canada [2]. Cannabis is the third most frequently used psychoactive substance in the province of Quebec, Canada, after alcohol and tobacco, with nearly 17% of Quebecers aged 15 or older reporting cannabis use according to a 2025 provincial report [3]. Cannabis users, particularly individuals with cannabis use disorder, are more likely to experience concomitant mental health issues (e.g., anxiety, depressive disorders), other substance use disorders (e.g., alcohol, cocaine), or behavioral addictions (e.g., gaming, gambling) compared to non-users [4, 5].

Behavioral addictions are addictions related to a behavior rather than a psychoactive substance [6]. Problematic internet use (PIU) is defined by excessive engagement in online activities (e.g., social media, hybrid games, video watching, information searching) [6, 7]. PIU can have various consequences, such as social isolation, family problems, psychological distress, and even suicide [8, 9]. In light of the marked increase in internet use since the COVID-19 pandemic [10], PIU is considered a public health priority by several stakeholders (e.g., government, researchers, health professionals).

PIU is often accompanied by significant burden and comorbidities [11–13], along with associated at-risk behaviors, such as cannabis use [7, 13–15]. Evidence supporting this association is seen in Lanthier-Labonté and colleagues’ [7] systematic review, which found that around 67% of the included studies reported a positive association between PIU and cannabis use among adolescents. A study conducted in Spain found that both cannabis abuse and dependence were associated with a higher risk of problematic video game use among 856 university students [5]. Lastly, according to Neilson and Lin [16] being under the influence of cannabis could contribute to extended periods of sedentary behavior. This may also be the case regarding prolonged use of the internet, as both behaviors generally occur while individuals are sitting or lying down (i.e., requiring little movement), and therefore using the internet may be regarded as a compatible activity while under the effect of cannabis.

While previous studies have mainly examined the relationship between cannabis use and internet use in adolescents [7, 17, 18], few have examined this relationship in adults, and to our knowledge, none have done so from a qualitative perspective. Therefore, the purpose of this study is to better understand the experiences of adult cannabis users regarding their non-medical consumption of cannabis (hereafter, cannabis consumption) and internet use.

## Method

### Study design

The data for this exploratory study with a qualitative component were obtained from a self-reported online questionnaire, completed via a web panel that was part of a larger cross-sectional correlational study [19]. Data collection took place from April 18th to May 15th, 2024. The questionnaire concluded with the following open-ended question: “In general, how would you describe the impact of cannabis consumption and internet use on your life (whether used together or separately)?” The present analysis is based on the participants’ responses to this open-ended question. The Consolidated Criteria for Reporting Qualitative Research (COREQ) [20] checklist was used to guide and ensure comprehensive and transparent reporting of this exploratory study with a qualitative component (see Supplementary Material).

### Recruitment and sampling

Participant recruitment was carried out using probabilistic (stratified random) sampling. An invitation email containing a personalized web link to the questionnaire was distributed via a firm specializing in surveys and web panels. The probabilistic sampling aimed to ensure that the Quebec population was represented in terms of age, sex and gender, language, and geographic region. To take part in this study, participants had to (1) be 18 years old or older, (2) have used cannabis (for non-medical purposes) at least once a week over the past 12 months, and (3) reside in the province of Quebec [19].

### Questionnaire design and content

The questionnaire, which included various tools that were validated in French and English, was divided into five sections and comprised 73 questions regarding (1) sociodemographic characteristics, (2) cannabis use and the use of other psychoactive substances, (3) internet use, (4) the association between cannabis consumption and internet use and the impacts of both, and (5) mental health (refer to Supplementary Material) [19]. To measure problematic cannabis use, participants who scored less than 3 (< 3) on the Cannabis Abuse Screening Test (CAST), a six-item screening scale that assesses cannabis abuse [21], were not considered at risk of problematic use. Participants scoring between 3 and 6 were considered at low risk, while those scoring 7 or above (≥ 7) were considered at high risk of problematic use. To measure PIU, participants who scored 30 or lower ( ≤ 30) on the Internet Addiction Test (IAT), an assessment tool designed to measure the degree of an individual’s internet use and its possible effects on their daily life [22, 23], were considered to show normal internet use. Participants scoring between 31 and 49 were considered to have a mild PIU. Participants scoring between 50 and 79 were considered to have a moderate PIU, while those scoring between 80 and 100 were considered to have a high PIU level. For the present study, scores of 50 and above were considered as presenting with PIU.

### Ethics statement

This study was conducted in accordance with the principled outlined by the Declaration of Helsinki. The research protocol was approved by the ethics committee of the Centre intégré universitaire de santé et de services sociaux (CIUSSS) de l’Estrie—Centre hospitalier universitaire de Sherbrooke (CHUS) on July 19th, 2023 (#2024-5139-CyberD-Cannabis). The data were anonymized, and all the participants provided free and informed written consent.

### Data analysis

The thematic analysis of the responses to the open-ended question aimed to provide a portrait of the participants’ experiences in their own words. An iterative coding approach was used to identify emerging and recurring themes in the participants’ discourse. A systematic thematic analysis was then conducted by JS and A-MA. During this phase, the thematic framework was enriched and refined. This version of the thematic framework was also revised and discussed with A-MA and MB. The thematic analysis, used to extract information from the data, was conducted by JS and A-MA according to the six-step procedure outlined by Braun and Clarke [24]. The first phase involved familiarization with the data, the second phase focused on generating initial codes, the third phase entailed searching for potential themes, the fourth phase consisted of reviewing the themes, the fifth phase involved defining and naming each theme, and finally, the sixth phase was dedicated to producing the analysis report. Some of the participants’ responses were written in French. To present them in this article, the responses were translated into English by the research team and reviewed by a professional translator.

## Results

### Sociodemographic characteristics

A total of 1,502 individuals participated in the study and completed the questionnaire, including the open-ended question. Of their responses, 114 were excluded due to random or void content. Therefore, a total of 1,388 responses to the open-ended question was subjected to thematic analysis for this article. Table 1 presents the sociodemographic profile of the participants who answered the open-ended question. The average age of the respondents was 43 years, and the sample consisted of 35% women and 65% men. The most common place of residence was in the urban Montreal area (33%). Most participants had obtained a college degree (42%), followed closely by those with a high school diploma (37%), and most respondents were employed (62%). The majority were shown to be at high risk of problematic cannabis use (58%), according to their score on the CAST (≥ 7). Additionally, 7% of the respondents demonstrated a moderate level of PIU, while 1% had high levels of PIU, as indicated by their IAT scores (50 to 79, and 80 to 100, respectively). The overall proportion of participants with PIU was 8%, according to the groupings mentioned in the Method section. The responses to the open-ended question varied in length and depth, ranging from a minimum of two words to a maximum of 269 words.

**Table 1:** Sociodemographic profile of the participants

	Participants (*N* = 1502)
Sex at birth	
Female	531 (35.4 %)
Male	971 (64.6 %)
Curent gender	
Women	515(34.3 %)
Men	973 (64.8 %)
Other gender	14 (0.9 %)
Age	42.6 (mean) and 14.02 (SD)
Region of Québec	
Bas-Saint-Laurent	34 (2.3 %)
Saguenay-Lac-Saint-Jean	59 (3.9 %)
Capitale-Nationale	111 (7.4 %)
Mauricie	57 (3.8 %)
Estrie	85 (5.7 %)
Montréal	495 (33.0 %)
Outaouais	78 (5.2 %)
Abitibi-Témiscamingue	19 (1.3 %)
Côte-Nord	12 (0.8 %)
Nord-du-Québec	7 (0.5 %)
Gaspésie/Îles-de-la-Madelaine	16 (1.1 %)
Chaudière-Appalaches	42 (2.8 %)
Laval	65 (4.3 %)
Lanaudière	92 (6.1 %)
Laurentides	97 (6.5 %)
Montérégie	195 (13.0 %)
Centre-du-Québec	38 (2.5 %)
Education	
Primary	27 (1.8 %)
High school	550 (36.6 %)
College	625 (41.6 %)
University certificates and diplomas	58 (3.9 %)
University bachelor’s degree	164 (10.9 %)
University master’s degree	71 (4.7 %)
University doctorate’s degree	7 (0.5 %)
Occupation	
Employee	936 (62.6 %)
Self-employed	105 (7.0 %)
Student	74 (4.9 %)
Retired	197 (13.2 %)
Unemployed	54 (3.6 %)
Social welfare	69 (4.6 %)
Other	60 (4.0 %)
Cannabis Abuse Screening Test (CAST)	
No risk (< 3)	162 (11.0%)
Low risk (3-6)	465 (31.0 %)
High risk(≥ 7):	874 (58.0 %)
Internet Addiction Test (IAT)	
Normal internet use (0-30)	1051 (70.0 %)
Mild PIU (31-49)	330 (22.0 %)
Moderate PIU (50-79)	105 (7.0 %)
High PIU (80-100)	15 (1.0 %)
Problematic internet use and problematic cannabis use	
CAST ≥ 7 and IAT≥ 50	93 (6.0%)

### Main themes

Four main themes emerged from the thematic analysis (see Table 2). The first theme concerns the absence of an impact of both behaviors on the participants’ lives as well as the lack of a visible link between their cannabis consumption and internet use (*no impact and no link*). The second theme explores the connection between cannabis consumption and internet use, (*Link between cannabis consumption and internet use*). The third theme addresses the impact of cannabis consumption or internet use on the participants’ physical and psychological health (*physical and psychological health*). This theme has eight sub-themes. The fourth theme addresses how cannabis consumption and internet use impact the participants’ lifestyle habits (*lifestyle habits*). This theme has seven sub-themes. The collected responses could be associated with more than one theme or sub-theme.

**Table 2:** Thematic tree of free-text responses regarding the impact(s) of cannabis consumption and internet use among adult cannabis users

Main themes	Sub-themes
1. No impact and no link	
2. Link between cannabis consumption and internet use	
3. Physical and psychological health	3.1 Relaxation effects 3.2 Pain management 3.3 Mood variations 3.4 Feelings of anxiety 3.5 Cognitive impacts 3.6 Changes in productivity 3.7 Use for distraction 3.8 Experiences of isolation
4. Lifestyle habits	4.1 Hobby 4.2 Social activity 4.3 Nutrition 4.4 Sleep 4.5 Financial considerations 4.6 Sexuality 4.7 Addiction

### No impact and no link

When asked about the impact of their cannabis consumption and internet use, around a third of the participants indicated that these behaviors had no effects on their lives. A smaller proportion of participants specified that they could not identify a link between their cannabis consumption and their internet use.

*No negative impact. Moderation is key. I don’t really see the link between cannabis and internet use.* (Participant 25268, F, age group 25–34)

*I see no impact. I use cannabis every day (generally in the evening)*,* and I go online every day*,* even without having smoked. I don’t go online because I’ve smoked cannabis.* (Participant 23460, F, age group 55–64)

Some participants expressed their thoughts on each of the two behaviors (cannabis consumption and internet use) independently, yet concluded they were unable to see how they could be related to one another.

*[…]I think that there are way too many people walking around high and driving high and I think that’s a big concern and a big danger to society. I also think that people are using social media and the internet to replace real relationships with their friends and family members and coworkers*, etc.,* I think it’s an interesting study but I’m not sure that the two things are corelated.* (Participant 4522, F, age group 25–34)

*I believe the two [cannabis consumption and internet use] aren’t connected in my life; I don’t see how my cannabis use is related to my activity on the internet. My cannabis use is to have fun with my friends*,* and my activity on the internet is to entertain myself when I’m alone.* (Participant 22161, F, age group 18–25)

### Link between cannabis consumption and internet use

A perceived link between cannabis consumption and internet use was observed participants’ responses to the open-ended question. The participants described how cannabis consumption led them to spend extended periods online, often engaging in specific online activities such as watching videos or movies, playing games, or browsing the web.

*I know that my cannabis use brings me to a neutral state in the evening*,* and I have a lot of difficulty putting down my tablet at those times. I know that if I reduced my use*,* I would be much more productive.* (Participant 4791, F, age group 35–44)

*If I consume [cannabis] and take my phone*,* if I make the mistake of going on games*,* I can stay on them for a very long time. That’s why I only take my phone in the evening.* (Participant 15072, F, age group 25–34)

*With cannabis*,* I go off on endless internet searches.* (Participant 5222, M, age group 25–34)

Some participants hypothesized as to why they felt they spent more time online while consuming, or after having consumed cannabis. For example, one participant mentioned a feeling of well-being after using cannabis, while others described feeling idle, prompting them to spend more time on the internet.

*I notice that I tend to want to use the internet more (e.g.*,* TikTok and Netflix) when I’ve used cannabis because of the sense of well-being I feel.* (Participant 18044, F, age group 18–24)

*I feel lazier when I use cannabis*,* so I spend more time on the internet.* (Participant 22086, F, 24–34)

*I think I use the Internet more when I consume cannabis because I zone out*,* but I do use the Internet a lot independently.* (Participant 3425, F, age group 25–34)

The increased time spent online was attributed by some participants to their losing track of time while consuming cannabis.

*Cannabis makes me stay on the internet longer. Loss of the sense of time. Additionally*,* one might make impulsive purchases for things that are not really needed.* (Participant 5268, F, age group 45–54)

*Cannabis makes me lose track of time when I’m on the internet.* (Participant 19464, F, age group 35–44)

*[When consuming cannabis] I stay longer on the internet*,* and time goes by… fast!* (Participant 21671, F, age group 35–44)

### Physical and psychological health

The participants mentioned that their physical and psychological health was impacted by their cannabis consumption and internet use. This third theme consists of eight sub-themes: (1) relaxation effects, (2) pain management, (3) mood variations, (4) feelings of anxiety, (5) cognitive impacts, (6) changes in productivity, (7) use for distraction, and (8) experiences of isolation.

#### Relaxation effects

Cannabis consumption and/or internet use were reported to give some participants a sense of relaxation and ease.

*In both cases*,* they bring me relaxation*,* just like someone who has a glass of wine*,* whether they are online or not.* (Participant 2863, M, age group 55–64)

This participant sees the two activities as fulfilling the same purpose and does not differentiate between them.

#### Pain management

Cannabis consumption was highlighted as beneficial for relieving various types of physical pain. It seems that this consumption helped the participants to cope with different physical ailments by reducing their suffering.

*I use cannabis to relax at the end of the day and also to relieve my pain related to [two health conditions]. Smoking helps me relax and fall asleep (my pain irritates me to the point of preventing me from sleeping).* (Participant 1511, F, age group 35–44)

#### Mood variations

Mood variations were another psychological impact of cannabis consumption and/or internet use. These mood changes were either described as positive or negative in the participants’ daily lives.

*When I consume too much cannabis*,* I become lethargic*,* I don’t want to do anything*,* I’m emotionally sensitive*,* I avoid people more*,* and my mood is unstable.* (Participant 22613, F, age group 35–44)

This participant views the two activities as serving the same function and does not distinguish between them.

*Cannabis and the internet bring joy to my life and help me relax.* (Participant 48, M, age group 35–44)

#### Feelings of anxiety

Changes in anxiety symptoms were reported in connection with cannabis consumption, with many describing these changes as positive because they reduced anxiety.

*It has an extremely positive impact; it allows me to think about something else*,* entertain myself*,* and relieve anxiety.* (Participant 7329, M, age group 35–44)

In contrast, others observed an increase in their anxiety.

*I don’t believe there is any major impact other than lack of sleep and increased anxiety.* (Participant 4386, M, age group 35–44)

#### Cognitive impacts

The participants also noted that cannabis consumption and/or internet use affect their cognitive functions. Many highlighted that these effects were positive, as they perceived an improvement in their memory, attention, or concentration.

*It helps me a lot to relax and focus on tasks at home that are more difficult for me due to my ADHD [attention deficit hyperactivity disorder].* (Participant 21488, F, age group 35–44)

*[When I consume cannabis]*,* it helps me stay focused on the task at hand. For example*,* responding to an important email without drifting off into 50 other searches related to ideas that come to mind.* (Participant 25176, M, age group 35–44)

Meanwhile, in other cases, the participants reported that these cognitive effects were negative, as they perceived a deterioration in their memory, attention, or concentration.

*[When I consume cannabis]*,* I experience some memory issues (but I don’t have any to begin with). It’s not really a problem for me.* (Participant 10655, F, age group 65–74)

One participant highlighted the importance of not using cannabis and the internet simultaneously.

*I try not to use them together. It’s a matter of concentration and fear of making mistakes or hurting someone.* (Participant 17174, M, age group 55–64)

#### Changes in productivity

Cannabis consumption and/or internet use were observed to affect productivity and motivation to carry out various daily tasks or activities.

*It slows me down and prevents me from doing everything I would like to.* (Participant 5625, F, age group 35–44)

Difficulties in stopping cannabis consumption and/or internet use were noted and often resulted in the postponement of important tasks.

*When I start smoking or using the internet*,* it’s very hard to stop. I try to limit it to the evening so that I eventually have to stop to go to bed*,* but it always results in me going to bed later than planned*,* as it’s hard to stop smoking and watching my shows when I’m online and/or smoking. I lose track of time and don’t do what I was supposed to do (housework*,* paperwork*,* errands…). After smoking or going online*,* I no longer feel like going out*,* seeing people*,* or doing anything*,* so I try to limit it to the evening to reduce the impacts.* (Participant 5766, F, age group 25–34)

This participant views the two activities as serving the same purpose and does not distinguish between them.

#### Use for distraction

Cannabis consumption and/or internet use were described as means of escape, helping individuals detach from daily worries.

*Both help me clear my mind*,* make me think about something else*,* and entertain me since I have stage 4 cancer. I manage to sleep much better and think about something other than cancer. In short*,* it gives me a break.* (Participant 11087, F, age group 45–54)

This participant does not make a distinction between the two activities, as they are used for the same purpose.

#### Experiences of isolation

Due to their cannabis consumption and/or internet use, some participants mentioned changes in their social life. Among these participants, some highlighted an increase in their isolation.

*[My cannabis consumption] has an impact on my social life. I tend to isolate myself to use it. I prefer to smoke alone (or with a friend I know well). I don’t like to smoke when I am with people I don’t know well*,* in person or on the phone*,* because it makes me uncomfortable. I have a paranoid (or very imaginative) tendency when I smoke*,* so I’ve avoided these awkward situations for a long time.* (Participant 22760, F, age group 55–64)

Meanwhile, other participants reported a decrease in their sense of isolation when they were consuming cannabis or using the internet. As a result, these two behaviors appear to help them address their feelings of loneliness.

*I’m quite solitary*,* so for both*,* they are essentials for me to fill my loneliness and express my emotions. [Without my cannabis consumption and internet use]*,* I would have little interest in anything. The world would feel like it was turned off if I didn’t consume cannabis and didn’t use the internet. It’s the way I’ve found to pass time.* (Participant 2557, F, age group 45–54)

This participant treats the two activities interchangeably, as they fulfill the same purpose.

### Lifestyle habits

As cannabis consumption and internet use can impact the participants’ daily lives, some participants described numerous lifestyle habits affected by these two behaviors, whether undertaken separately or together. The fourth theme consists of seven sub-themes: (1) hobbies, (2) social activity, (3) nutrition, (4) sleep, (5) financial considerations, (6) sexuality, and (7) addiction.

#### Hobby

Cannabis consumption and/or internet use were described as sources of entertainment and hobbies.

*They add to my life without taking up too much space. Both are good forms of entertainment.* (Participant 29729, M, age group 45–54)

*I use cannabis and the internet to relax and entertain myself during appropriate moments of my day.* (Participant 10209, F, age group 35–44)

#### Social activity

Some participants mentioned that they consume cannabis and/or use the internet in the presence of friends or family members.

*No impact*,* as cannabis consumption is only for pleasure and with friends. In those moments*,* there is no room for the internet unless we are looking up specific things for our personal knowledge (e.g.*,* word definitions*,* characters in movies).* (Participant 16035, F, age group 55–64)

#### Nutrition

Some participants mentioned that diet and appetite were impacted by cannabis consumption.

*I think cannabis has little impact on my life except in terms of my diet. I eat much more when I smoke and probably watch more movies and series.* (Participant 10199, F, age group 25–34)

*Eating too much before bed*,* which negatively affects my sleep and overall health.* (Participant 12455, M, age group 35–44)

#### Sleep

Cannabis consumption and/or internet use were found to affect the participants’ sleeping habits. Some participants reported a positive impact from cannabis consumption, noting improved sleep.

*I take [cannabis] oil for deeper sleep. Besides this desired effect*,* there is no other impact.* (Participant 14197, M, age group 55–64)

However, a negative impact on sleep was also noted as a consequence of cannabis consumption and/or internet use.

*I often find that I spend too much time on my phone. Smoking cannabis makes me go to bed late*,* which leads to other problems related to lack of sleep.* (Participant 28749, F, age group 35–44)

#### Financial considerations

Finances were also identified as an aspect of lifestyle habits affected by cannabis consumption and/or internet use. The majority of the participants mentioned a loss of money, either through the purchase of cannabis or online spending.

*It makes me angry and ruins my life because I waste my time*,* my money*,* and especially my health.* (Participant 27458, F, age group 25–34)

*The only negative impact of cannabis consumption is its effect on my budget.* (Participant 2573, M, age 35–44)

However, one participant mentioned a positive impact on their finances because they could earn money online.

*It’s easy for me to spend a few hours on Facebook or YouTube. Sometimes*,* I fill out surveys to get some pocket money.* (Participant 18378, F, age group 55–64)

#### Sexuality

Cannabis consumption was noted to lead to sexual excitement, which prompted participants to watch pornography online.

*[When I use cannabis]*,* I spend a lot of time watching pornography online*,* which interferes with other household tasks. It’s an addiction.* (Participant 1668, M, age group 45–54)

Thus, participants seem to acknowledge that their cannabis consumption affects not only their sexuality but also their internet use, leading to an increase in the consumption of online pornography.

#### Addiction

Some participants highlighted that their cannabis consumption and/or internet use have become addictions. They shared testimonies illustrating these addictions and their effects on their daily lives.

*I think I am addicted; if I go long without both*,* I start to feel withdrawal.* (Participant 3075, F, age group 18–24)

*I have been a user of cannabis since adolescence*,* so for me*,* it’s called addiction*,* but I am very aware of it.* (Participant 9402, M, age group 55–64)

On the other hand, the difficulty in stopping these behaviors was mentioned.

*After four and a half years of therapy*,* I am much better and have a much better capacity for self-observation. However*,* I notice that anxiety and performance anxiety create a vicious cycle with internet use. It is the only addiction I still cannot manage. I recognize it and believe that I will one day succeed.* (Participant 3277, F, age group 25–34)

## Discussion

The purpose of this study was to better understand the experiences of adult cannabis users regarding both their cannabis and internet use. The open-ended nature of the question allowed the participants to develop on the perceived impact cannabis consumption and internet use on their lives, either together or separately. This format led to a wide range of responses. The sample consisted of individuals with different patterns of cannabis consumption (ranging from once a week to multiple times in a day) and internet use. This diversity in the participants’ profiles likely represents this variability in their responses. The proportion of participants with PIU in our sample was estimated at 8%, which is comparable to the rates reported among the general population in other countries [25].

About a third of respondents did not perceive that their cannabis consumption or internet use had any effect on their lives, or did not see a connection between the two behaviors. Many participants reporting no impact, also reported their use of cannabis and the internet was “moderated” or “under control”, while some declared having issues with one of the behaviors, but not the other. Other participants described how cannabis consumption and internet use served different purposes in their lives, and therefore they did not perceive a link between the two. The perception of no impact could be related to the increasingly normalized view of cannabis consumption in Canadian society, both pre-and post-legalization [26, 27]. According to Duff et al. [26], who conducted semi-structured interviews among adult regular cannabis users in four major cities in Canada, while individuals regarded cannabis use as a “as a normal and largely harmless practice” (p.276), they emphasized the importance of controlling personal use, such that it was “neither bothering anyone nor interfering with one’s responsibilities” (p.278). Similarly, as the internet is more present than ever in daily life, therefore, it is possible individuals are unable to perceive a direct impact of the internet on their life when they see its use as “normal”.

However, most participants were able to identify the impacts that their cannabis consumption and/or internet use had on their lives, including their psychological and physical health or their lifestyle habits. Some participants observed an impact of cannabis consumption on their internet use, such that cannabis consumption led them to stay online for longer. Similarly, Neilson and Lin [16], reported that regular or occasional cannabis users were at greater odds of engaging in high risk (> 35 h per week) sedentary behavior during leisure time, such as browsing the internet and watching online videos. Additionally, a study conducted among substance users in southern Italy found that cannabis users and cocaine users spend more time online daily than those who use alcohol or opioids [28]. While in the previous cross-sectional studies it is impossible to establish a direction to the association between the time spent online and cannabis consumption, the participants who endorsed this theme perceived it is the effect of cannabis consumption that prompts an increased use of the internet.

Some participants described the beneficial effects of their cannabis consumption and/or internet use on their psychological and physical well-being. They mentioned that these activities helped them relax, experience positive emotions, improve their mental skills, reduce feelings of isolation, increase productivity, and alleviate physical pain. The positive feelings experienced while they were engaging in both activities, as reported by some participants of our study, are consistent with participants’ discourses in a previous qualitative study [29], which prospectively studied the course of cannabis use and dependence of adult cannabis users over three years. Liebregts et al. [29] described many of their participants used cannabis either to relax, or to enhance the experience of a leisure activity. For example, some individuals described playing video games and simultaneously consuming cannabis to enhance the experience and moderate emotional reactions [29]. The positive perception of cannabis consumption as a social activity, mentioned by some participants, can also be observed in Liebregts et al.’s [29] results, in which over half of participants (and mainly those classified as non-cannabis-dependent) considered cannabis primarily as a social activity. This claim has also been examined quantitatively by Okey et al. [30], who reported that frequent solitary cannabis consumption was associated to more negative consequences than social use of cannabis.

Conversely, other participants reported that their cannabis consumption and/or internet use had negative effects on their psychological health. They noted that these behaviors amplified their anxious feelings, promoted procrastination and passivity, intensified their loneliness, and impaired their mental faculties. The impact of cannabis consumption on sexuality was also addressed by a few participants, such that they enjoyed watching pornography online while under the effect of cannabis. Previous studies have also examined the effect of cannabis on sexual experiences, both quantitatively [31, 32] and qualitatively [33], with overall results indicating cannabis may increase sexual desire, and amplify or enhance sensations during sexual activity (including masturbation). This may explain why certain participants turn to pornography online. Furthermore, the analysis revealed that these two behaviors also influenced the respondents’ daily lives, for some positively, while for others negatively, regarding their financial situation, sleep quality, eating habits, social relationships, and leisure activities. Further exploration into what drives positive versus negative effects related to cannabis use could help inform harm reduction recommendations for regular cannabis users.

Overall, the results of the present study reflect a lot of the heterogeneity found in the literature on this topic [7, 34]. While some studies report a positive association between cannabis use and internet use, others find no such association, which was also observed in the participants’ perspectives in the present study [7]. Interestingly, the cited systematic review found that all studies using a measure of cannabis dependence or addiction (such as the CAST, which was employed in the present study) reported a positive association between PIU and cannabis use. This highlights the importance of ongoing evaluation and refinement of the concept of cannabis dependence to improve understanding of the harms associated to cannabis use and related addictive behaviors.

### Limitations

This research has certain limitations. The first limitation concerns the variability in the length of the responses obtained, which made it difficult to construct a complete picture of the participants’ cannabis consumption and internet use habits. A second limitation could be related to the placement of the open-ended question at the end of the questionnaire, which may have led participants to respond quickly without taking the time to reflect deeply on their answers. Similarly, since the responses to the open-ended question varied in length from one participant to another, it was sometimes difficult to determine how internet use was defined by a participant. As such, it is not possible to know what the participants considered to be internet use (e.g., watching videos, purchasing public transportation tickets, accessing online bank accounts). This ambiguity in definition may influence how certain results are interpreted, particularly for participants who reported no association between cannabis consumption and internet use. The third limitation involves the use of a self-report questionnaire, which may have increased social desirability bias. Finally, the fourth limitation concerns the data collection method, which did not allow follow-up questioning with the participants.

### Strengths

Despite these limitations, the 1,388 responses collected provide a broad range of experiences from individuals who consume cannabis and use the internet. Additionally, this study specifically focuses on the adult population, allowing for a better understanding of the association between cannabis consumption and internet use within this age group. The qualitative analysis of responses to an open-ended question offers a deeper understanding of the context behind the answers provided in the questionnaire. It creates a detailed portrait of the participants’ experiences, which cannot be achieved solely through responses to closed-ended questions. Finally, this type of research is valuable for accessing a large amount of qualitative data regarding the experiences of a significant proportion of individuals [35, 36].

### Future research directions

For future research, conducting semi-structured interviews to gain a deeper understanding of the themes identified by the participants would be beneficial. Additionally, investigating the association between these two behaviors within diverse populations in terms of age, sex, gender, and other sociodemographic factors would be pertinent. The current literature primarily focuses on adolescent cannabis users, a population identified as being at high risk for developing PIU [7, 17, 18]. Continued research with various social groups would not only enhance the understanding of the specificities of each group but also help develop more tailored public policies and interventions for different social contexts. Future qualitative research should place greater focus on exploring the interaction between cannabis consumption and internet use, as this aspect was not explicitly emphasized in the open-ended question.

## Conclusion

Some participants indicated that their cannabis consumption and internet use had no impact on their lives or that they did not perceive any relationship between them. Conversely, it was found that these behaviors influenced the psychological and physical health of most participants as well as various aspects of their lifestyle. Among those who noted the impacts of these behaviors on their lives, some described in detail the role that cannabis consumption plays in their internet use. This study provides preliminary insights into the participants’ experiences and reflects the aspects they considered essential to share regarding their cannabis consumption and internet use. This study highlights the relevance of further research to better comprehend whether a relationship and interaction between these two behaviors exists, and what the extent of this relationship is.

## Supplementary Information

Supplementary Material 1.

Supplementary Material 2.

## Data Availability

All data and material used for this study are available from the corresponding author upon reasonable request.

## Abbreviations

Cannabis Abuse Screening Test (CAST): A six-item screening scale that assesses cannabis abuse
Internet Addiction Test (IAT): A twenty-item assessment tool designed to measure the degree of an individual’s internet usage and its possible effects on their daily life
Consolidated Criteria for Reporting Qualitative Research (COREQ): Quality appraisal tool to ensure a comprehensive and transparent reporting of qualitative research
