# Supplementary material for: Cannabis consumption and problematic internet use: analysis of a free-text response collected through a self-reported online questionnaire
Source: BMC Psychol. 2026 Mar 24;14:640. doi: 10.1186/s40359-026-04419-3 (PMC13134161; doi:10.1186/s40359-026-04419-3)
Supplement: Supplementary file 2 — Supplementary Material 2. [file 40359_2026_4419_MOESM2_ESM.docx]

**QUESTIONNAIRE ON CYBERADDICTION AND CANNABIS USE-English Version**

- - 1. **LANGUAGE**

[POSER À TOUS]

[MENTION SIMPLE]

- - 1. **LANG**

Would you prefer to complete the survey in English or French?

| **Libellé** | **Valeur** | **Attribut** | **Terminaison** |
| --- | --- | --- | --- |
| Français | FR |  |  |
| English | EN |  |  |

- - 1. **SECTION 1 – WEIGHING**
       - 1. **PROV + Variables standard de régions**

In which province or territory do you reside?

| **Libellé** | **Valeur** | **Attribut** | **Terminaison** |
| --- | --- | --- | --- |
| British Columbia | BC |  | TERMINER |
| Alberta | AB |  | TERMINER |
| Saskatchewan | SK |  | TERMINER |
| Manitoba | MB |  | TERMINER |
| Ontario | ON |  | TERMINER |
| Quebec | QC |  |  |
| New Brunswick | NB |  | TERMINER |
| Nova Scotia | NS |  | TERMINER |
| Prince Edward Island | PE |  | TERMINER |
| Newfoundland and Labrador | NF |  | TERMINER |
| Northwest Territories | NT |  | TERMINER |
| Yukon | YK |  | TERMINER |
| Nunavut | NU |  | TERMINER |
| I live outside of Canada | 97 |  | TERMINER |

- - 1. **AGENUM**

How old are you?

__________ years old

| **Libellé** | **Valeur** | **Attribut** | **Terminaison** |
| --- | --- | --- | --- |
| I prefer not to answer. | 99 |  | TERMINER |

**SEXE1**

What was the **sex assigned to you at birth?**

*Sex refers to the legally assigned gender on the birth certificate, which may differ from your current gender.*

| **Libellé** | **Valeur** | **Attribut** | **Terminaison** |
| --- | --- | --- | --- |
| Masculin  Male | 1 |  |  |
| Féminin  Female | 2 |  |  |

- - 1. **SEXE2**

### What is your gender?

### By gender, we mean your current gender, which may differ from the sex assigned at birth or from that recorded in legal documents.

| **Libellé** | **Valeur** | **Attribut** | **Terminaison** |
| --- | --- | --- | --- |
| Homme  Man | 1 |  |  |
| Femme  Woman | 2 |  |  |
| Je préfère identifier mon genre  I prefer to identify my gender | 96 | F |  |

- - 1. **SEX3**

Which term best describes your **current gender identity?**

Note: A **cisgender** man or woman is a person whose sex assigned at birth is identical to their current gender identity (e.g., a person assigned female at birth who identifies as female).

*Select all choices that apply.*

| **Libellé** | **Valeur** | **Attribut** | **Terminaison** |
| --- | --- | --- | --- |
| Cisgender man | 1 |  |  |
| Cisgender woman | 2 |  |  |
| Trans person | 3 |  |  |
| Non-binary person | 4 |  |  |
| Gender creative / non-conforming person | 5 |  |  |
| If other, please specify: _________________________ | 96 | O |  |
| I prefer not to answer. | 99 | X |  |

**QOR1**

Which term(s) best describes **your current sexual orientation?**

*Select all choices that apply.*

Heterosexual (1)

Gay (2)

Lesbian (3)

Bisexual (4)

Pansexual (5)

Queer (6)

Asexual (7)

Two-spirited (8)

Questioning (9)

I prefer to identify my current sexual orientation (please specify): _________________________ (96) – O

I prefer not to answer. (99) – X

- - 1. **FOY1**

Including yourself, how many people (adults and children) live in your household?

*Please enter your answer here.*

___ person(s)

| **Libellé** | **Valeur** | **Attribut** | **Terminaison** |
| --- | --- | --- | --- |
| Only one (myself) | 1 |  |  |
| I prefer not to answer. | 99 |  | TERMINER |

- - 1. **FOY2**

Of these ('FOY1') people in your household, how many are children under the age of 18?

*Please enter your answer here.*

___ child(ren)

| **Libellé** | **Valeur** | **Attribut** | **Terminaison** |
| --- | --- | --- | --- |
| None | 0 |  |  |
| I prefer not to answer. | 99 |  | TERMINER |

- - 1. **LANGU**

What is the language you first learned at home as a child that you still understand?

| **Libellé** | **Valeur** | **Attribut** | **Terminaison** |
| --- | --- | --- | --- |
| French | 1 |  |  |
| English | 2 |  |  |
| A language other than English or French | 3 |  |  |
| I prefer not to answer. | 99 |  | TERMINER |

**SCOL**

What is the highest level of education you have received?

| **Libellé** | **Valeur** | **Attribut** | **Terminaison** |
| --- | --- | --- | --- |
| Primary (7 years or less) | 1 |  |  |
| Secondary (general or vocational training [8 to 12 years]) | 2 |  |  |
| College (pre-university training, technical training, certificates, attestations, or diplomas [13 to 15 years]) | 3 |  |  |
| University – certificates and diplomas | 4 |  |  |
| University first cycle – Bachelor’s degree (including classical course) | 5 |  |  |
| University second cycle – Master’s degree | 6 |  |  |
| University third cycle – PhD | 7 |  |  |
| I prefer not to answer. | 99 |  | TERMINER |

- - 1. **SECTION 2 – ELIGIBILITY CRITERIA**

**Q1**

**In the past 12 months**, have you used any of the following substances?

*Select all choices that apply.*

Yes (1)

No (2)

Alcoholic beverages (e.g., beer, wine, or spirits) (1)

Tobacco (e.g., cigarette, cigar, pipe, hookah, or chewing tobacco) (2)

Cannabis (e.g., marijuana, joint, herb, or hash) (3)

Drugs (other than cannabis; e.g., cocaine, heroin, or other opioids) (5)

None of the above (97)

I prefer not to answer (99)

**Q2**

**In the past 12 months**, have you used cannabis…

***For medical purposes*** *(i.e., by obtaining a medical document from an authorized health professional when the latter judges that a cannabis product would be beneficial for the patient's health)?*

***For nonmedical purposes*** *(i.e., for a range of nonmedical reasons, such as for social, amusement, spiritual, lifestyle, or other nonmedical reasons)?*

*[Sources:*

*National Institute of Public Health of Quebec. (2016). Canadian medical cannabis regime.*

*Government of Canada. (2022). 2022 Canadian Cannabis Survey.]*

For medical purposes (1)

For nonmedical purposes (2)

For medical and nonmedical purposes (3)

I don't know (98)

I prefer not to answer (99)

**Q3**

**In the past 12 months**, how often have you used cannabis for nonmedical purposes?

Less than 1 day per month (1)

1 day per month (2)

2 to 3 days per month (3)

1 to 2 days per week (4)

3 to 4 days per week (5)

5 to 6 days per week (6)

Every day (7)

Several times a day (Specify the number of times: _____) (96) – O (Numérique : Min 1, Max 30)

I don't know (98)

I prefer not to answer (99)

**Q4**

**In the past 12 months**, how often have you used cannabis for medical purposes?

Less than 1 day per month (1)

1 day per month (2)

2 to 3 days per month (3)

1 to 2 days per week (4)

3 to 4 days per week (5)

5 to 6 days per week (6)

Every day (7)

Several times a day (Specify the number of times: _____) (8) – O (Numérique : Min 1, Max 30)

I don't know (98)

I prefer not to answer (99)

- - 1. **SECTION 3 – SOCIODEMOGRAPHIC PROFILE**

### **QSCTDEMO**

The next questions are about your socio-demographic profile. All of your answers will remain strictly anonymous and confidential.

- - 1. **POSTAL3**

Please enter the first 3 characters of your postal code.

__________________________

| **Libellé** | **Valeur** | **Attribut** | **Terminaison** |
| --- | --- | --- | --- |
| I would prefer not to answer | 99 |  |  |

**ETHN**

Which of the following choices best describes you?

*Select all choices that apply.*

White (1)

Indigenous (First Nations, Métis, or Inuk/Inuit) (2)

Latin American (3)

Middle Eastern / North African (4)

Black (5)

South Asian (6)

Southeast Asian (7)

West Asian / Central Asian (8)

Chinese (9)

Filipino (10)

Japanese (11)

Korean (12)

Identify my ethnic origin: ___ (96)

I prefer not to answer (99)

**Q5**

Do you identify yourself as an **Aboriginal person of Canada** (First Nations, Inuit, or Metis)?

Yes (1)

No (2)

I prefer not to answer. (99)

**Q6**

What is your current marital status?

Single (1)

Common law (2)

Married (3)

Separated or divorced (4)

Widowed (5)

Other (96) – F

I prefer not to answer. (99)

**Q7**

Which living situation applies to you now?

I live alone. (1)

I live with my spouse(s). (2)

I live with my spouse and children. (3)

I live alone with children. (4)

I live with my parents. (5)

I live with one or more adults who is or are not my spouse or parent. (6)

If other, please specify: ________________________ (96) – O

I prefer not to answer. (99)

**Q8**

How would you describe your current **main occupation**?

Salaried employee (1)

Self-employed (2)

Student (3)

Retired (4)

Unemployed (5)

On social assistance (6)

If other, please specify: ________________________ (96) – O

I prefer not to answer. (99)

**Q9A**

What was your **ANNUAL PERSONAL INCOME before taxes in 2023**?

$1–19,999 (2)

$20,000–29,999 (3)

$30,000–39,999 (4)

$40,000–49,999 (5)

$50,000–59,999 (6)

$60,000–69,999 (7)

$70,000–79,999 (8)

$80 ,000–89,999 (9)

$90,000–99,999 (10)

$100,000–109,999 (11)

$110,000–119,000 (12)

$120,000–129,000 (13)
$130,000–139,000 (14)

$140,000–149,000 (15)

$150,000 or more (16)

I prefer not to answer. (99)

**Q9B**

What was your **ANNUAL HOUSEHOLD INCOME before taxes in 2023?**

$1–19,999 (2)

$20,000–29,999 (3)

$30,000–39,999 (4)

$40,000–49,999 (5)

$50,000–59,999 (6)

$60,000–69,999 (7)

$70,000–79,999 (8)

$80 ,000–89,999 (9)

$90,000–99,999 (10)

$100,000–109,999 (11)

$110,000–119,000 (12)

$120,000–129,000 (13)
$130,000–139,000 (14)

$140,000–149,000 (15)

$150,000 or more (16)

I prefer not to answer. (99)

- - 1. **SECTION 4 – PROFILE OF CANNABIS CONSUMPTION AND CONSUMPTION OF OTHER PYSHCOACTIVE SUBSTANCES**

**QINF1(CAN)**

The next questions relate to your **cannabis use.**

"In this study, we use the term cannabis. This term includes marijuana (pot, weed, grass), hashish (hash), liquid (cannabis oil) or solid (shatter, budder, wax) extracts or concentrates, and any other product made from the cannabis plant but does not include synthetic cannabinoids (Spice, K2, Yucatan Fire).

Synthetic cannabinoids are products created in the laboratory, often sprayed or sprinkled on a plant substrate, mimicking certain effects of cannabis.

When we ask about using cannabis, we are referring to smoking it, vaping it, consuming it in an edible, or using it in any other way."

*Source: Health Canada.(2017). Enquête canadienne sur le cannabis (ECC), MainInto. Adapted by the Institut de la Statistique du Québec (ISQ).*

**Q10**

What age category were you in when you **first used cannabis**?

Under 12 years (1)

12 to 15 years (2)

16 to 17 years (3)

18 to 20 years (4)

21 to 24 years (5)

25 to 29 years (6)

30 to 34 years (7)

35 years and older (8)

I don't know (98)

I prefer not to answer (99)

**Q11**

**In the past 12 months**, have you used any of the following **methods** to consume cannabis?

The **method** refers to how cannabis is consumed.

Yes (1)

No (2)

I don't know (98)

I prefer not to answer (99)

Smoked it in a joint, water pipe, pipe, or cigar (1)

Inhaled it by "dabbing," which includes inhalation from a knife, needle, hot nail, or bottle ("lead") (2)

Inhaled it in the form of e-liquid by vaping (e.g., with an electronic cigarette) (3)

Inhaled it by vaporization (e.g., with a stationary or portable vaporizer) (4)

Ate it in a food product (e.g., brownies, cakes, cookies, or candy) (5)

Drank it (e.g., in tea, a soft drink, or alcohol) (6)

Ingested it in a pill or capsule (7)

Consumed it in the form of oral drops or using an oral atomizer (e.g., spray) (8)

**Q12**

**In the past 12 months,** have you used a **method other** than those mentioned above to consume cannabis?

The **method** refers to how cannabis is consumed.

Yes, please specify: _______ (1) - O

No (2)

I don't know (3)

I prefer not to answer (4)

**Q13A**

**In the past 12 months**, have you used any of the following **forms** of cannabis?

The **form** corresponds to the cannabis product being consumed.

*Select all choices that apply.*

Dried flowers, leaves, or buds (1)

Hashish or skuff (kif or pollen) (2)

Liquid extracts or concentrates (e.g., cannabis or hashish oil or extract, such as rosin or distillate, in an oil cartridge or disposable vaporizer pen) (3)

Solid extracts or concentrates (e.g., dabs, shatter, budder, or wax) , **except for hashish** (4)

Food products (5)

Drinks (6)

Other form (specify): ________ (96) – O

**Q13B**

**Over the past 12 months**, in what form did you use cannabis **most often**?

The **form** corresponds to the cannabis product being consumed.

Dried flowers, leaves, cocottes, or buds (1)

Hashish or skuff (kif or pollen) (2)

Liquid extracts or concentrates (e.g., cannabis or hashish oil or extract, such as rosin or distillate, in an oil cartridge or disposable vaporizer pen) (3)

Solid extracts or concentrates (e.g., dabs, shatter, budder, or wax) , except for hashish (4)

Food products (5)

Drinks (6)

Other form (specify): ________ (96) – O

**Q15**

**In the past 12 months,** have you consumed any **synthetic cannabinoids** (e.g., Spice, K2, or Yucatan Fire)?

*Synthetic cannabinoids are products created in the laboratory, often sprayed or sprinkled on a plant substrate, with the aim of mimicking certain effects of cannabis. These products may have other names, such as AK-47, Mr. Happy, Scooby Snax, Kush, or Kronic, and generally contain molecules such as CP-55940, JWH-018, or HU-210.*

Yes (1)

No (2)

I don't know (3)

I prefer not to answer (4)

**Q17**

For what reason(s) have you used cannabis in the **past 12 months**?

*Select all choices that apply.*

To relax or unwind (1)

To experiment or see what it does (2)

To feel "buzzed," "stoned" or "high" (3)

To help you with your sleep (4)

To help you with your feelings or emotions (5)

To decrease or increase the effect of another substance / psychoactive substance (6)

To avoid withdrawal (7)

For pleasure (8)

To have a good time with friends, socialize, or facilitate relationships (i.e., be less shy) (9)

To increase particular sensations (e.g., sexuality, the sound of music in festive environments, or the taste of food) (10)

For ceremonial use or other spiritual purposes (11)

To broaden one's level of consciousness (i.e., explore alternative ways of thinking and seeing the world) (12)

To treat a health problem or relieve symptoms (13)

Other reason (specify): ________ (96) – O

**Q19**

**During the past 12 months**, did you have a medical prescription from a doctor authorizing you to consume cannabis?

Yes (1)

No (2)

I don't know (3)

I prefer not to answer (4)

**Q20**

**Over the past 12 months,** how often have you used cannabis just before or during a work or study day (on site or remotely)?

Never (1)

Sometimes (2)

Often (3)

Always or almost always (4)

Does not apply; I was not working or studying (5)

I don't know (6)

I prefer not to answer (7)

**Q21**

**Over the past 12 months,** what general effect has your cannabis use had on ...?

Positive effect (1)

No effect (2)

Negative effect (3)

Positive and negative effects (4)

I don't know (98)

I prefer not to answer (99)

Your friendships or social life (1)

Your physical health (2)

Your physical mobility (3)

Your mental health (4)

Your family life (5)

Your life as a couple (6)

Your work or studies (7)

Your quality of life (8)

**Cannabis Abuse Screening Test (CAST)**

**Q22**

In the last 12 months…

LISTE - COLONNES

1: Never

2: Rarely

3: From time to time

4: Fairly often

5: Very often

ÉNONCÉS – LIGNES

Have you smoked cannabis within 2 to 4 hours after your wake-up time? (1)

Have you smoked cannabis when you were alone? (2)

Have you had memory problems when you smoked cannabis? (3)

Have friends or members of your family told you that you ought to reduce your cannabis use? (4)

Have you tried to reduce or stop your cannabis use without succeeding? (5)

Have you had problems because of your use of cannabis (argument, fight, accident, bad result at school, etc.)? (6)

**Alcohol, Smoking and Substance Involvement Screening Test (ASSIST)**

**Q23**

In your life which of the following substances **(other than cannabis)** **have you ever used (non-medical use only)**?

LISTE - COLONNES

No (1)

Yes (2)

I prefer not to answer. (99)

ÉNONCÉS – LIGNES

Tobacco products (cigarettes, chewing tobacco, cigars, etc.) (1)

Alcoholic beverages (beer, wine, spirits, etc.) (2)

Cocaine (coke, crack, etc.) (3)

Amphetamine-type stimulants (speed, meth, ecstasy, etc.) (4)

Inhalants (nitrous, glue, petrol, paint thinner, etc.) (5)

Sedatives and sleeping pills (diazepam, alprazolam, flunitrazepam, midazolam, etc.) (6)

Hallucinogens (LSD, acid, mushrooms, trips, ketamine, etc.) (7)

Opioids (heroin, morphine, methadone, buprenorphine, codeine, etc.) (8)

Other - specify: ___________(96)-O

**QPROBE**

Not even when you were in school/during your studies?

LISTE - COLONNES

No (1)

Yes (2)

I prefer not to answer. (99)

ÉNONCÉS – LIGNES

Tobacco products (cigarettes, chewing tobacco, cigars, etc.) (1)

Alcoholic beverages (beer, wine, spirits, etc.) (2)

Cocaine (coke, crack, etc.) (3)

Amphetamine-type stimulants (speed, meth, ecstasy, etc.) (4)

Inhalants (nitrous, glue, petrol, paint thinner, etc.) (5)

Sedatives and sleeping pills (diazepam, alprazolam, flunitrazepam, midazolam, etc.) (6)

Hallucinogens (LSD, acid, mushrooms, trips, ketamine, etc.) (7)

Opioids (heroin, morphine, methadone, buprenorphine, codeine, etc.) (8)

Other - specify: ___________(96)-O

**Q24**

In the **past 3 months** how often have you **used the substances you mentioned**?

LISTE - COLONNES

Never (1)

Once or twice (2)

Monthly (3)

Weekly (4)

Daily/almost daily(5)

**Q25**

During **the past 3 months** how often have you had a **strong desire or urge to use [drug]?**

LISTE - COLONNES

Never (1)

Once or twice (2)

Monthly (3)

Weekly (4)

Daily/almost daily (5)

**Q26**

During the past 3 months how often has your use of [drug] led **to health, social, legal or financial problems**?

LISTE - COLONNES

Never (1)

Once or twice (2)

Monthly (3)

Weekly (4)

Daily/almost daily (5)

**Q27**

During the **past 3 months** how often have you **failed to do what was normally expected of you** because of your use of [drug]?

LISTE - COLONNES

Never (1)

Once or twice (2)

Monthly (3)

Weekly (4)

Daily/almost daily (5)

**Q28**

Has a friend or relative or anyone else ever expressed **concern about your use of** [drug]?

LISTE - COLONNES

No, never (1)

Yes, in the past three months (2)

Yes, but not in the last three months (3)

**Q29**

Have you ever tried to cut **down or stop using** [drug] **but failed**?

LISTE - COLONNES

No, never (1)

Yes, in the past three months (2)

Yes, but not in the last three months (3)

**Q30**

Have you ever used any drug **by injection (NON-MEDICAL USE ONLY)**?

LISTE - COLONNES

No, never (1)

Yes, in the past three months (2)

Yes, but not in the last three months (3)

**Q31**

**Over the past 12 months,** how often have you used any of the following substances in combination with cannabis (i.e., at the same time as cannabis)?

Never (1)

Rarely (2)

Sometimes (3)

Often (4)

Always (5)

I don't know (8)

I prefer not to answer (9)

Alcohol (1)

**Tobacco or nicotine** in any of its forms (either smoked or in an electronic cigarette; either mixed with cannabis or not) (2)

**Prescription opiates or opioids** (either prescribed for you or not; e.g., oxycodone, Dilaudid®, morphine, Demerol, Fentanyl, or medications containing codeine) (3)

**Prescription stimulants** (either prescribed for you or not; e.g., Ritalin®, Concerta®, Adderall®, Dexedrine®, or Vyvanse®) (4)

**Prescription sedatives or tranquilizers** (either prescribed for you or not; e.g., "Benzos," such as diazepam, lorazepam, Valium®, Ativan®, alprazolam, Xanax, clonazepam, or Rivotril®) (5)

**Antihistamine, antitussive, decongestant, or anti-nausea medication** (either prescribed for you or not, e.g., Gravol®, Bénadryl®, Dorm-aide®, Sleep-EZEMD, DM syrup, Sudafed®) (6)

**Other substance / psychoactive substance** (96)

- - 1. **SECTION 5 – INTERNET USE PROFILE**

**QINF2 (AC)**

The following questions are about **your access to the Internet**.

**Exclude business and school-related use.**

**Q32**

Do you have access to the Internet at home?

Yes(1)
No(2)
I don’t know (97)

**Q33**

Do you have access to the internet at work and/or at school?

Yes(1)
No(2)
I don’t know (97)

**Q34**

Do you have access to the Internet through a mobile data plan for personal use?

*A data plan allows users to access the Internet from anywhere through a wireless handheld device, such as smartphone or tablet.*

Yes(1)
No(2)
I don’t know (97)

**Q35**

During the past three months, what devices did you use to access the Internet?

*Select all that apply.*

Smartphone (1)

Laptop or netbook (2)

Tablet (3)

Desktop computer (4)

Media streaming device (5)

SmartTV (6)

Smartwatch (7)

Video game console (8)

Internet-connected wearable smart device (9)

Virtual reality device (10)

Smart vehicle device(11)

If other devices, please specify : _______ (96) – O

**QINF3**

The following questions are about **technology and social connections.**

**Q36**

**In the past month**, how often did you do the following activities?

LISTE - COLONNES

Everyday (1)

A few times a week (2)

Once a week (3)

Two or three times a month (4)

Once a month (5)

Not in the past month (6)

Not applicable (99)

ÉNONCÉS – LIGNES

Use the Internet to communicate with friends or keep up with their activities (1)

Get together in-person with friends outside of work or school (2)

Use the Internet to communicate with relatives or family members or keep up with their activities (3)

Get together in-person with relatives or family members (4)

**Q37**

**In the past 12 months**, excluding time spent on schoolwork, your job, and other obligations, how often did you use screens to:

LISTE - COLONNES

Never (1)

Once (2)

Sometimes (3)

Every month (4)

Once or twice a week (5)

Several times a week (6)

Every day (7)

Je préfère ne pas répondre (99)

ÉNONCÉS – LIGNES

Game / play (1)

Maintain / participate in / develop your social network (Facebook, Instagram, Habouki, Snapchat, TikTok, Twitter(X), etc.) (2)

Talk (chat room, instant messaging, texting) (3)

Watch entertainment videos, YouTubers, or influencers (4)

Watch video games, eSports or Fantasy sports (Twitch, etc.) (5)

Get informed (blogs, forums, medias, Wikipedia, etc.) (6)

Create content for the Internet (7)

Date people (Bumble, Grindr, Tinder, Zoosk, etc.) (8)

Watch series, movies or TV (Netflix, Apple TV+, etc.) (9)

Shop online (Amazon, eBay, Ali Express, Etsy, etc.) (10)

Autre(s) activité(s) (précisez) : __________________ (96) - O

**Q39**

In the **last 12 months**, how many hours on average **per day** did you spent on screen activities (**including** time spent on schoolwork, your job, and other obligations)?

Less than 1 hour (1)

1 to 3 hours (2)

4 to 6 hours (3)

7 to 9 hours (4)

10 to 12 hours (5)

13 to 15 hours (6)

16 or more (7)

**Q40**

In the **last 12 months**, how many hours on average **per day** did you spent on screen activities, **excluding** time spent on schoolwork, your job, and other obligations (i.e., on a day off or weekend)?

Less than 1 hour (1)

1 to 3 hours (2)

4 to 6 hours (3)

7 to 9 hours (4)

10 to 12 hours (5)

13 to 15 hours (6)

**INTERNET ADDICTION TEST (IAT)**

**Q41**

**Over the past month,** how often have the following statements applied to you?

LISTE - COLONNES

1 : Not Applicable

2 : Rarely

3 : Occasionally

4 : Frequently

5 : Often

6 : Always

ÉNONCÉS – LIGNES

1. How often do you find that you stay online longer than you intended?

2. How often do you neglect household chores to spend more time online?

3. How often do you prefer the excitement of the Internet to intimacy with your partner?

4. How often do you form new relationships with fellow online users?

5. How often do others in your life complain to you about the amount of time you spend online?

6. How often do your grades or schoolwork suffer because of the amount of time you spend online?

7. How often do you check your email before something else that you need to do?

8. How often does your job performance or productivity suffer because of the Internet?

9. How often do you become defensive or secretive when anyone asks you what you do online?

10. How often do you block out disturbing thoughts about your life with soothing thoughts of the Internet?

11. How often do you find yourself anticipating when you will go online again?

12. How often do you fear that life without the Internet would be boring, empty, and joyless?

13. How often do you snap, yell, or act annoyed if someone bothers you while you are online?

14. How often do you lose sleep due to being online?

15. How often do you feel preoccupied with the Internet when off-line, or fantasize about being online?

16. How often do you find yourself saying "just a few more minutes" when online?

17. How often do you try to cut down the amount of time you spend online and fail?

18. How often do you try to hide how long you've been online?

19. How often do you choose to spend more time online over going out with others?

20. How often do you feel depressed, moody, or nervous when you are off-line, which goes away once you are back online?

**Q42**

Do you play **online** video games?

Yes (1)

No (2) 🡪 SAUTER le questionnaire IGDT-10

**Internet Gaming Disorder Test (IGDT-10)**

**Q43**

Please read the statements below regarding **online video gaming.**

This questionnaire concerns **ONLINE VIDEO GAMES,** but the words "games", “gaming” and "play" are used for simplicity. Please indicate on a scale of "Never" to "Often" how much, and how often, these statements apply to you over the **last 12 months.**

|  | Never  (1) | Sometimes  (2) | Often  (3) |
| --- | --- | --- | --- |
| 1. When you were not playing, how often have you fantasized about gaming, thought of previous gaming sessions, and/or anticipated the next game? | ○ | ○ | ○ |
| 2. How often have you felt restless, irritable, anxious and/or sad when you were unable to play or played less than usual? | ○ | ○ | ○ |
| 3. Have you ever felt the need to play more often or played for longer periods to feel that you have played enough? | ○ | ○ | ○ |
| 4. Have you ever unsuccessfully tried to reduce the time spent on gaming? | ○ | ○ | ○ |
| 5. Have you ever played games rather than meet your friends or participate in hobbies and pastimes that you used to enjoy before? | ○ | ○ | ○ |
| 6. Have you played a lot despite negative consequences (for instance losing sleep, not being able to do well in school or work, having arguments with your family or friends, and/or neglecting important duties)? | ○ | ○ | ○ |
| 7. Have you tried to keep your family, friends or other important people from knowing how much you were gaming or have you lied to them regarding your gaming? | ○ | ○ | ○ |
| 8. Have you played to relieve a negative mood (for instance helplessness, guilt, or anxiety)? | ○ | ○ | ○ |
| 9. Have you risked or lost a significant relationship because of gaming? | ○ | ○ | ○ |
| 10. Have you ever jeopardized your school or work performance because of gaming? | ○ | ○ | ○ |

- - 1. **SECTION 6 – SOCIAL NETWORK USE**

**Q44**

Please select **all the social media** that you have viewed **at least once a week in the past 12 months**.

| **COMMUNICATION** |  |  |  |
| --- | --- | --- | --- |
| Messenger (Meta) | **1** |  |  |
| WhatsApp | **2** |  |  |
| Snapchat | **3** |  |  |
| Telegram | **4** |  |  |
| Viber | **5** |  |  |
| WeChat | **6** |  |  |
| BeReal | **7** |  |  |
| **CONTENT** |  |  |  |
| YouTube | **8** |  |  |
| Instagram | **9** |  |  |
| Facebook | **10** |  |  |
| TikTok | **11** |  |  |
| Twitter (X) | **12** |  |  |
| Threads | **13** |  |  |
| Pinterest | **14** |  |  |
| Reddit | **15** |  |  |
| Tumblr | **16** |  |  |
| Twitch | **17** |  |  |
| Discord | **18** |  |  |
| Mastodon | **19** |  |  |
| **MEETINGS** |  |  |  |
| Tinder | **20** |  |  |
| Bumble | **21** |  |  |
| Hinge | **22** |  |  |
| Grindr | **23** |  |  |
| LinkedIn | **24** |  |  |
| Facebook Dating | **25** |  |  |
| Other social media | **96** |  |  |
| None of these | **97** |  |  |

**Q45**

Which **social media** do you **use most often** among those you selected above?

*Please rank these platforms from 1 to 5, with 1 being the one you use the most.*

**Bergen Social Media Addiction Scale**

**Q46**

Over the past year, how often have you...

LISTE - COLONNES

Very rarely (1)

Rarely (2)

Sometimes (3)

Often (4)

Very often (5)

ÉNONCÉS – LIGNES

Spent a lot of time thinking about social media or planning how to use it. (1)

Felt an urge to use social media more and more. (2)

Used social media in order to forget about personal problems. (3)

Tried to cut down on the use of social media without success. (4)

Become restless or troubled if you are prohibited from using social media. (5)

Used social media so much that it has had a negative impact on your job/studies. (6)

**Online Fear of Missing Out Inventory (On-FOMO)**

**Q47**

Please answer the following questions.

LISTE - COLONNES

Has nothing to do with me (1)

Has a little to do with me (2)

Something to do with me (3)

Has a lot to do with me (4)

ÉNONCÉS – LIGNES

When I see on a social network that a friend is somewhere where I 0.71

wanted to go too, I feel bad. (1)

I get annoyed when my friends do not tag me in posts. (2)

I get sad to learn from posts that my friends went to events and I wasn't invited. (3)

Often, I feel sad seeing on social networks that people are happier than I am. (4)

I feel distant from people when I see them happy in posts. (5)

I get annoyed when my posts do not get many likes and/or comments. (6)

I only post photos or videos that I know my friends will like. (7)

I need people to like or comment on my posts. (8)

I am indifferent to my friends' reactions to my posts. (9)

I would like to have more likes and/or comments on my posts. (10)

I get anxious when my cell phone does not have internet signal. (11)

If I do not have access to social networks, I think of ways to get connected. (12)

I think a lot about social networks when I do not have access to them. (13)

I get restless when I cannot access social networks. (14)

I usually feel irritated by staying disconnected from social networks too long. (15)

When I'm on social networks, I forget my problems. (16)

My family and friends complain that I spend a lot of time connected to social networks. (17)

When I start checking for updates, I find it hard to leave social networks. (18)

In social situations, I pay more attention to my cell phone than to my friends. (19)

I am late to appointments because of social network use. (19)

- - 1. **SECTION 7 – LINK BETWEEN CANNABIS CONSUMPTION AND INTERNET USE AND ITS IMPACTS**

**Q48**

**In the past 12 months**, how often have you used cannabis while using the Internet?

Never when I use the Internet (1)

Once when I use the Internet (2)

Rarely when I use the Internet (3)

Sometimes when I use the Internet (4)

Often when I use the Internet (5)

Always when I use the Internet (6)

I don't know (96)

I prefer not to answer (99)

**Q50**

What online activity(ies) do you engage in while using cannabis?

*Select all choices that apply.*

Gaming/playing (1)

Maintaining / participating in / developing your social network(s) (e.g., Facebook, Instagram, Snapchat, TikTok, or Twitter/X) (2)

Chatting (e.g., instant messaging, texting, or participating in a chat room) (3)

Watching entertainment, YouTuber, or influencer videos (4)

Watching video games or electronic sports (e.g., eSports or Twitch) (5)

Informing yourself (e.g., via blogs, forums, media, or Wikipedia) (6)

Creating content to distribute on the Internet (7)

Meeting people (e.g., on Bumble, Grindr, Tinder, or Zoosk) (8)

Watching series, films, or television (e.g., on Netflix or Apple TV+) (9)

Shopping online (e.g., on Amazon, eBay, Ali Express, or Etsy) (10)

Other online activity(ies) (specify): __________________ (96) – O

**Q51**

**In the past 12 months,** have you used cannabis in any of the following situations?

Yes (1)

No (2)

Before using the Internet (1)

While using the Internet (2)

After using the Internet (3)

**Q52**

In your opinion, how does your **cannabis use impact** your **use of the Internet**?

I spend more time on the Internet because of my cannabis use (1)

I spend less time on the Internet because of my cannabis use (2)

No impact (3)

I prefer not to answer (99)

**Q53**

In your opinion, how does your **use of the Internet impact** your **cannabis consumption**?

I use cannabis more often because of my Internet use (1)

I use cannabis less often because of my Internet use (2)

No impact (3)

I prefer not to answer (99)

**Q54**

Do you think you have or have ever had an Internet use disorder and/or a cannabis use disorder (*i.e., use/usage that is risky, excessive, or impulsive in nature, resulting in adverse life consequences, particularly physical, emotional, social, or functional impairment)*?

Neither (1)

Internet use disorder (2)

Cannabis use disorder (3)

Both Internet use disorder and cannabis use disorder (4)

I prefer not to answer (99)

**Q55**

Have you ever sought **help regarding your cannabis consumption?**

Yes (1)

No (2)

I prefer not to answer. (99)

**Q56**

Which of the **resources** below have you ever consulted in your lifetime related to your **cannabis consumption?**

*Select all that apply.*

Family doctor (1)

Nurse (2)

Addiction counsellor (3)

Specialist (e.g., psychiatrist) (4)

Psychologist (5)

Social worker (6)

Helpline (e.g., Drugs, Help and Referral, Connex Ontario, or Addiction Help Line) (7)

Self-help group (e.g., Narcotics Anonymous) (8)

Religious or spiritual counsellor (9)

Friend (10)

Family (11)

Spouse (12)

Self-management resources (reading books, consulting Internet forums, videos, or podcasts on the subject) (13)

If other, please specify: ________________________ (96) – O

**Q57**

How often have you consulted each of these resources **in the past year in relation to your cannabis use?**

Once (1)

A few times a year (less than once a month) (2)

Once or twice a month (3)

Once a week (4)

More than once a week (5)

I prefer not to answer (99)

**Q58**

How useful do you think each of these resources were?

Completely helpful (1)

Somewhat helpful (2)

Neither helpful nor useless (3)

Somewhat useless (4)

Completely useless (5)

I prefer not to answer. (99)

**Q59**

Among the following activities, please select the one(s) associated with your problematic Internet use.

*Select all choices that apply.*

Gaming/playing (1)

Maintaining / participating in / developing your social network(s) (e.g., Facebook, Instagram, Snapchat, TikTok, or Twitter/X) (2)

Chatting (e.g., instant messaging, texting, or participating in a chat room) (3)

Watching entertainment, YouTuber, or influencer videos (4)

Watching video games or electronic sports (e.g., eSports or Twitch) (5)

Informing yourself (e.g., via blogs, forums, media, or Wikipedia) (6)

Creating content to distribute on the Internet (7)

Meeting people (e.g., on Bumble, Grindr, Tinder, or Zoosk) (8)

Watching series, films, or television (e.g., on Netflix or Apple TV+) (9)

Shopping online (e.g., on Amazon, eBay, Ali Express, or Etsy) (10)

Other online activity(ies) (specify): __________________ (96) - O

**Q60**

Have you ever sought **help regarding your Internet use?**

Yes (1)

No (2)

I prefer not to answer. (99)

**Q61**

Which of the **resources** below have you ever consulted in your lifetime related to your **Internet use?**

*Select all that apply.*

Family doctor (1)

Nurse (2)

Addiction counsellor (3)

Specialist (e.g., psychiatrist) (4)

Psychologist (5)

Social worker (6)

Helpline (e.g., Drugs, Help and Referral, Connex Ontario, or Addiction Help Line) (7)

Self-help group (e.g., Narcotics Anonymous) (8)

Religious or spiritual counsellor (9)

Friend (10)

Family (11)

Spouse (12)

Self-management resources (reading books, consulting Internet forums, videos, or podcasts on the subject) (13)

If other, please specify: ________________________ (96) – O

**Q61B**

How often have you consulted each of these resources **in the past year in relation to your Internet use?**

Once (1)

A few times a year (less than once a month) (2)

Once or twice a month (3)

Once a week (4)

More than once a week (5)

I prefer not to answer (99)

**Q62**

How useful do you think each of these resources were?

Completely helpful (1)

Somewhat helpful (2)

Neither helpful nor useless (3)

Somewhat useless (4)

Completely useless (5)

I prefer not to answer. (99)

**SECTION 8 – MENTAL HEALTH PROFILE**

Mental Health and Access to Care Survey (MHACS), 2022

**Q63**

In general, how is your mental health?

Would you say:

1: Excellent

2: Very good

3: Good

4: Fair

5: Poor

98: I don’t know

99: I prefer not to answer.

**Q64**

Compared to one year ago, how would you say your mental health is now?

1: Much better now than 1 year ago

2: Somewhat better now (than 1 year ago)

3: About the same as 1 year ago

4: Somewhat worse now (than 1 year ago)

5: Much worse now (than 1 year ago)

98: I don’t know

99: I prefer not to answer.

**Q65**

Do you have a **family doctor?**

Yes (1)

No (2)

I don’t know. (98)

I prefer not to answer. (99)

**Q66**

Have you ever been or are you currently being followed by a doctor or another healthcare professional (including addiction interveners) for one or more of the following health problems?

Yes (1)

No (2)

I prefer not to answer (99)

Mental health disorders (e.g., depression, anxiety, or bipolar disorder) (1)

Alcohol-related problems (2)

Problems related to cannabis use (3)

Problems related to the use of substances / psychoactive substances (other than cannabis) (4)

Problems related to problematic Internet use (5)

**Q67**

Have you been diagnosed with a **mood disorder** such as depression, bipolar disorder, mania, or dysthymia?

Yes (1)

No (2)

I don’t know. (98)

I prefer not to answer. (99)

**Q68**

What kind of mood disorder have you been diagnosed with?

1: Depression

2: Bipolar disorder (manic depression)

3: Mania

4: Dysthymia

96: If other, please specify: ________________________– O

98: I don’t know.

99: I prefer not to answer.

**Q69**

Have you ever been diagnosed with **an anxiety disorder** such as a phobia, obsessive-compulsive disorder or a panic disorder?

Yes (1)

No (2)

I don’t know. (98)

I prefer not to answer. (99)

**Q70**

What anxiety disorder(s) have you been diagnosed with?

*Select all that apply.*

Generalized anxiety disorder (GAD) (1)

Social anxiety disorder / social phobia (2)

Specific phobia (3)

Obsessive-compulsive disorder (OCD) (4)

Panic disorder (5)

Other (specify): _______ (96) - O

I don’t know (98)

I prefer not to answer (99)

**Generalized Anxiety Disorder-7 (GAD-7)**

**Q71**

Over the **last two weeks**, how often have you been bothered by the following problems?

LISTE - COLONNES

1: Not at all

2: Several days

3: More than half the days

4: Nearly every day

ÉNONCÉS – LIGNES

1: Feeling nervous, anxious, or on edge

2: Not being able to stop or control worrying

3: Worrying too much about different things

4 : Trouble relaxing

5: Being so restless that it is hard to sit still

6: Becoming easily annoyed or irritable

7: Feeling afraid, as if something awful might happen

**Patient Health Questionnaire-8 (PHQ-8)**

**Q72**

Over the **last 2 weeks,** how often have you been bothered by any of the following problems?

LISTE - COLONNES

1: Not at all

2: Several days

3: More than half the days

4: Nearly every day

ÉNONCÉS – LIGNES

1: Little interest or pleasure in doing things

2: Feeling down, depressed, or hopeless

3: Trouble falling or staying asleep, or sleeping too much

4: Feeling tired or having little energy

5: Poor appetite or overeating

6: Feeling bad about yourself or that you are a failure or have let yourself or your family down

7: Trouble concentrating on things, such as reading the newspaper or watching television

8: Moving or speaking so slowly that other people could have noticed. Or the opposite being so fidgety or restless that you have been moving around a lot more than usual.

**Q73**

In general, how would you describe the impacts of cannabis use and Internet use (together or separately) on your life?

*Do not hesitate to share any details you think may be relevant. They will be useful to us.*

**QCONTACT1**

Would you like to be contacted again to participate in Phase 2 of this project (individual interviews)?

Yes (1)

No (2)

### QCONTACT2

Please provide your contact information, so that we can get back to you:

(1) Name:

(2) First name:

(3) Email address:

(4) Phone number:
